# Discovery of Bile Acid Derivatives as Potent ACE2 Activators by Virtual Screening and Essential Dynamics

**DOI:** 10.1021/acs.jcim.1c01126

**Published:** 2021-12-16

**Authors:** Bianca Fiorillo, Silvia Marchianò, Federica Moraca, Valentina Sepe, Adriana Carino, Pasquale Rapacciuolo, Michele Biagioli, Vittorio Limongelli, Angela Zampella, Bruno Catalanotti, Stefano Fiorucci

**Affiliations:** † Department of Pharmacy, Università di Napoli “Federico II”, Via D. Montesano, 49, I-80131 Napoli, Italy; ‡ Department of Medicine and Surgery, Università di Perugia School of Medicine, Piazza L. Severi, I-06132 Perugia, Italy; § Net4Science S.r.l., University “Magna Græcia” of Catanzaro, Campus Universitario “S. Venuta”, I-88100 Catanzaro, Italy; ∥ Faculty of Biomedical Sciences, Euler Institute, Università della Svizzera italiana (USI), via G. Buffi 13, CH-6900 Lugano, Switzerland

## Abstract

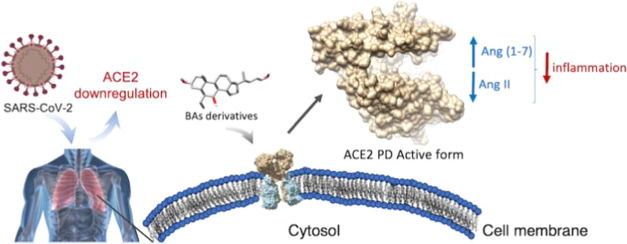

The angiotensin-converting enzyme II (ACE2) is a key molecular player in the regulation of vessel contraction, inflammation, and reduction of oxidative stress. In addition, ACE2 has assumed a prominent role in the fight against the COVID-19 pandemic-causing virus SARS-CoV-2, as it is the very first receptor in the host of the viral spike protein. The binding of the spike protein to ACE2 triggers a cascade of events that eventually leads the virus to enter the host cell and initiate its life cycle. At the same time, SARS-CoV-2 infection downregulates ACE2 expression especially in the lung, altering the biochemical signals regulated by the enzyme and contributing to the poor clinical prognosis characterizing the late stage of the COVID-19 disease. Despite its important biological role, a very limited number of ACE2 activators are known. Here, using a combined in silico and experimental approach, we show that ursodeoxycholic acid (UDCA) derivatives work as ACE2 activators. In detail, we have identified two potent ACE2 ligands, BAR107 and BAR708, through a docking virtual screening campaign and elucidated their mechanism of action from essential dynamics of the enzyme observed during microsecond molecular dynamics calculations. The *in silico* results were confirmed by *in vitro* pharmacological assays with the newly identified compounds showing ACE2 activity comparable to that of DIZE, the most potent ACE2 activator known so far. Our work provides structural insight into ACE2/ligand-binding interaction useful for the design of compounds with therapeutic potential against SARS-CoV-2 infection, inflammation, and other ACE2-related diseases.

## Introduction

The angiotensin-converting enzyme II (ACE2) is a type I transmembrane mono-carboxypeptidase that recently reached the headlines for its role as a human receptor for the SARS-CoV-2 virus, which is responsible for the outbreak of COVID-19.^1^ More specifically, using the spike (S) glycoprotein, SARS-CoV-2 binds to ACE2 on the surface of epithelial cells as the first step of the viral replication strategy. The S protein is composed of two subunits: the S1 subunit that contains a receptor-binding domain (RBD) that engages with the host cell receptor ACE2, and the S2 subunit that mediates the fusion with the host cell membrane.^2^ The strong binding of the S protein to ACE2 through the RBD activates the proteolytic cleavage of the S2 subunit by the transmembrane serine protease 2 (TMPRSS2), enabling the entry of the virus into cells and promoting the viral replication and cell-to-cell transmission and the consequent spread of the coronavirus throughout the host.^3^

ACE2 is expressed in several tissues, such as the lung, arteries, heart, kidney, and gastrointestinal tract,^4−6^ playing a key role in blood pressure regulation, fluid and electrolyte balance, and cardiac/renal function. The biological role of ACE2 has been extensively investigated in the last decade, and it is essentially related to the extracellular enzymatic conversion of vasoconstrictor peptide angiotensin (Ang) II to the vasodilatory peptide Ang (1–7). In the renin-angiotensin system (RAS), angiotensin-converting enzyme (ACE) converts Ang I to Ang II, which signals through the Ang II type I receptor (AT1R), resulting in vasoconstriction, increased fluid retention, cardiac and vascular hypertrophy, oxidative stress, and tissue fibrosis.^7−10^ Conversely, ACE2 hydrolyzes Ang II into Ang (1–7), thus reducing the availability of Ang II and preventing the proinflammatory and vasoconstrictive effects through AT1R signaling. In addition, Ang (1–7) activates the Mas G-protein coupled receptor (MasR), promoting vasodilation and reduction of oxidative stress and inflammation due to the release of nitric oxide, prostaglandin E2, and bradykinin.^11−13^ In particular, Ang (1–7) counteracts leukocyte migration, cytokine expression and release, and fibrogenic pathways.^11^ Consistent with this view, ACE2 deficiency promotes vascular inflammation and atherosclerosis in ApoE knockout mice by increasing the expression of vascular cells adhesion molecules (VCAM), cytokines, chemokines, and MMP.^14^ In contrast, the activation of the ACE2/Ang (1–7)/MasR axis decreases the expression of inflammatory mediators including TNFα, IL-1β, IL-6, and MCP-1 and increases the expression of the anti-inflammatory cytokine IL-10.^14−16^ Additional studies have reported the relationship between the ACE2/Ang (1–7)/MasR axis and cancer growth suggesting the therapeutic potential of the activation of ACE2 in cancer inhibition.^17^

SARS-CoV-2 cell invasion induces downregulation of ACE2 expression that results in the alteration of the physiological balance between Ang II and Ang (1–7), increasing the Ang II-mediated RAS signaling and, on the other hand, depleting the protective effects mediated by ACE2/Ang (1–7)/MasR axis. These phenomena have been related to specific clinical conditions in COVID-19 patients, such as the cytokine storm and coagulopathy.^18,19^ Therefore, the strengthening of the ACE2 activity might be a potential approach to limit the damage due to the excessive inflammatory response that leads to the worst complications in the COVID-19 disease.^20^

Despite the therapeutic potential of ACE2 activators, only a limited number of such agents are currently available.^21,22^ Among these is diminazene aceturate (DIZE), an anti-trypanosomiasis veterinary drug that also works as an ACE2 activator,^23^ leading to protective effects in experimental models of hypertension,^24^ myocardial infarction,^25^ liver injury,^26^ and kidney disease.^27^ Moreover, DIZE attenuates inflammation in an NF-κB-dependent manner^28^ and plays protective effects in ischemia-induced cardiac pathophysiology.^25^ For these reasons, the use of DIZE as a protective agent for COVID-19 patients has been proposed,^29^ although it demonstrated cytotoxic side effects at therapeutic doses.^30^

Several three-dimensional ACE2 structures have been released in the Protein Data Bank (PDB) so far, paving the way to structure-based drug discovery approaches for the identification of candidate drugs that might bind with high affinity and selectivity to the target. Considering the lack of safe and druggable ACE2 activators, we engaged a structure-based virtual screening for the discovery of novel natural compounds able to activate ACE2. Prompted by our previous studies showing that endogenous bile acids (BAs) might interfere with S Spike/ACE2 interaction, here, we report a series of ursodeoxycholic acid (UDCA) derivatives as novel ACE2 activators. Our combined in silico and experimental approach allows delineating the mechanism of action of the novel compounds that involves a peculiar ligand-induced conformational behavior of the enzyme. The two-best compounds of the series, BAR107 and BAR708, promote ACE2 activation comparable to that elicited by DIZE; therefore, these agents have therapeutic potential in the prevention and treatment of coronavirus-mediated infection and inflammation as well as in other diseases related to the disfunction of RAS/Mas signaling.

The results of our studies suggest that compounds with a steroidal scaffold can be useful to develop dual-activity drug candidates, combining the ability to reduce Spike/ACE2 interaction and the protective effect deriving from the activation of the ACE2/Ang (1– 7)/MasR axis.

## Methods

### Computational Studies

#### Virtual Screening

The crystal structure of the open apo form of homo sapiens ACE2 (PDB ID 1R42)^31^ was downloaded from the Protein Data Bank website. The disordered segment of the collectrin homology domain and water molecules were removed. The receptor was treated with the Protein Preparation Wizard tool^32^ implemented in Maestro ver. 11.8^32^ to assign bond orders, to add hydrogen atoms, adjust disulfide bonds, and assign residues protonation state at pH 7.4.

Virtual screening (VS) was performed on an in-house library of 67 bile acids (BAs), 10 natural and 57 semisynthetic derivatives, enriched with previously identified ACE2 activators, hydroxyzine, minithixen, and DIZE.^21^ Chemical/physical properties of all of the 67 compounds were calculated with QikProp tool ver. 5.8^32^ and are shown in the plots of Figure S1 in the Supporting Information. Since even minor structural changes of steroids can produce potential biological activities, we build our in-house BAs library to include compounds sharing a 17-carbon-atom skeleton composed of four fused rings, which form the typical steroidal scaffold. They vary from one another in the position and name of the substituent groups. The steroidal carbons hydrogens that have been replaced in our *in-house* library are: (i) those in positions 3 and 7, which have been replaced with a hydroxyl group in both different configurations (α and β); (ii) the hydrogen at C7, which has been replaced with an ethyl group in both configurations (α and β); (iii) finally, the C24 has been substituted with different polar and apolar groups. Docking calculations were performed in a box including the hinge-bending region of ACE2 on the Protein Data Bank deposited structure of ACE2 in the open conformation (PDB ID 1R42), according to previous reports on the discovery of ACE2 activators.^21^ The VS procedure was carried out with the AutoDock4.2.6 suite^33^ and the Raccoon2 graphical interface^34^ using the Lamarckian genetic algorithm (LGA). The VS protocol adopted was the same described in our previous work.^35^ To further assess our docking protocol, re-docking calculations were performed on the potent ACE2 inhibitor MLN-4760 in the ACE2 binding site (PDB ID 1R4L). Given the presence of a Zn^2+^ ion coordinating the ligand, the improved AutoDock4(Zn) force field was used for the calculation.^36^ As shown in Figure S2 in the Supporting Information, AutoDock4(Zn) well reproduced the experimental binding pose.

The receptor was submitted to the AutoGrid4 tool, which calculated interaction grids, considering the two ligands and receptor-atom types through the definition of a cubic box of 46 × 46 × 46 Å. Subsequently, for each grid, AutoDock4 calculated interaction energies (ADscore) that express the affinity of a given ligand for the receptor. All of the images were rendered using UCSF Chimera.^37^

#### Molecular Dynamics (MD)

MD simulations of apo ACE2 and ACE2 in complex with BAR708 and BAR107 were performed with the CUDA version of the AMBER18 suite^38,39^ using the Amber *ff*14*SB*^40^ to treat the protein, while ligands charges were computed using the restrained electrostatic potential (RESP) fitting procedure.^41^ First, the ligand ESP was calculated through the Gaussian16 package^42^ using the 6-31G* basis set at Hartree–Fock level of theory. Then, RESP charges and the ligand force field parameters were obtained from the two-stage fitting procedure using Antechamber^43^ and the general amber force field (GAFF2) parameters.^44^ The system was then immersed in a preequilibrated octahedral box of TIP3P water molecules, and the system was neutralized. The system was then minimized and successively equilibrated in a multistep procedure as previously described.^35^ Specifically, each system was minimized in four steps using the energy gradient convergence criterion set to 0.01 kcal/mol Å^2^ involving: (i) 5000 minimization steps (2500 with the steepest descent and 2500 with the conjugate gradient) of only hydrogen atoms; (ii) 20 000 minimization steps (10 000 with the steepest descent and 10 000 with the conjugate gradient) of water and hydrogen atoms, keeping the solute restrained; (iii) 50 000 minimization steps (25 000 with the steepest descent and 25 000 with the conjugate gradient) of only the side chains of the protein, water, and hydrogen atoms; (iv) 100 000 (50 000 with the steepest descent and 50 000 with the conjugate gradient) of complete minimization. Successively, water molecules, ions, and protein side chains were thermally equilibrated in three steps: (i) 5 ns of NVT equilibration with the Langevin thermostat by gradually heating from 0 to 300 K, while gradually rescaling solute restraints from a force constant of 10 to 1 kcal/mol Å^2^; (ii) 5 ns of NPT equilibration at 1 atm with the Berendsen thermostat by gradually rescaling restraints from 1.0 to 0.1 kcal/mol Å^2^; and (iii) 5 ns of NPT equilibration with no restraints. Finally, three independent MD production runs of 500 ns each were performed for each system using a timestep of 2 fs. The SHAKE algorithm was used for those bonds containing hydrogen atoms in conjunction with periodic boundary conditions at constant pressure and temperature, particle mesh Ewald (PME)^45^ for the treatment of long-range electrostatic interactions and a cutoff of 10 Å for nonbonded interactions.

#### Principal Component Analysis

**Figure 1:**
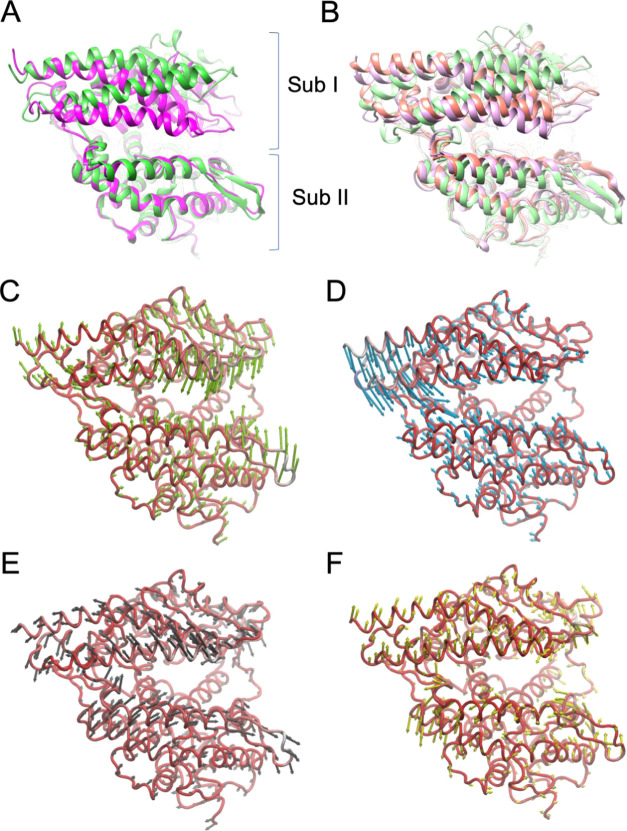
Dynamic behavior of native ACE2. Superimposition on the Sub II protein backbone atoms between: (A) the X-ray structures of the open apo ACE2 (PDB ID 1R42) (green cartoon) and the closed state of ACE2 complexed with the potent inhibitor MLN-4760 (PDB ID 1R4L) (magenta cartoon); (B) the most populated three clusters of apo ACE2 obtained after 1.5 μs MD simulation: Cluster0 (32% population, light-violet cartoon), Cluster1 (20% population, light-green cartoon), and Cluster 2 (18% population, light-red cartoon); (C–F) porcupine plots of the first four eigenvectors (PC1–4) identified from the PCA analysis after 1.5 μ MD simulations of apo ACE2. Protein backbones are represented as red ribbons, while arrows indicate the direction of the prominent motions and the length represents the magnitude of the corresponding eigenvalue.

The principal component analysis (PCA)^46^ of apo ACE2 and ACE2 complexed with BAR708 and BAR107 was carried out using the CPPTRAJ module^47^ of the AMBER18 Suite. First, the overall 1.5 μs of MD trajectories of each system was stripped of solvent and ions. Then, to take into account the internal dynamics of ACE2, global rotational/translational motions of the protein were removed by fitting the stripped trajectories to the protein heavy atoms of the first MD frame. This allowed us to generate the average structure of the protein of each system, which was used as the reference structure for the PCA analysis. Finally, we have generated the coordinate covariance matrix and diagonalized it, thus obtaining the first four principal components (PCs) as eigenvectors and eigenvalues. The pseudotrajectory of the protein motion was then imported and visualized into the Normal Mode Wizard GUI (NMWiz)^48^ of VMD, to generate the porcupine plot of each motion, with the arrows representing the magnitude and direction of the eigenvectors (Figure 1C–F). All of the images were rendered using UCSF Chimera.^37^

#### ACE2 Inhibitor Screening Assay Kit

The ACE2 Inhibitor Screening Assay Kit (BPS Bioscience Catalogue # 79923) measures the exopeptidase activity of ACE2. It utilizes the ability of an active ACE2 to cleave a Fluorogenic Substrate to release a free fluorophore that can be easily quantified using a fluorescence microplate reader. In the presence of an ACE2 specific inhibitor, the enzyme loses its peptidase activity, which results in a decrease of fluorescence intensity, whereas in the presence of an activator, an increase in fluorescence is observed. DIZE was used as a positive control of enzymatic activity.

Briefly, the purified ACE2 is thawed and diluted in ACE2 buffer to the concentration of 0.5 ng/μL ACE2. This Enzyme Solution (20 μL) is added to each well of a 96-well plate, designated as “Positive Control” and “Test Inhibitor”. Conversely, the wells designated as “Blank” contain only the ACE2 buffer (20 μL). Next, 5 μL of Test Inhibitor solution are added to each well designated Test Inhibitor. For the wells labeled Positive Control and Blank, add 5 μL of the Inhibitor buffer (10% DMSO in water). Finally, 25 μL of ACE2 Fluorogenic Substrate is added to all wells and the reaction is incubated at room temperature for 60 min, protected from direct light. After the incubation, the fluorescence intensity of the samples (λ excitation = 555 nm; λ emission = 585 nm) is measured in a FluoStar Omega microplate reader.

#### ACE2-SARS-CoV-2 Spike Inhibitor Screening Assay Kit

The ACE2-SARS-CoV-2 Spike Inhibitor Screening Assay Kit (BPS Bioscience Catalogue #79936) is designed for screening and profiling inhibitors of this interaction. This kit contains purified ACE2 and SARS-CoV-2 Spike (RBD)-Fc proteins, HRP-labeled anti-mouse-Fc region antibody, and assay buffers. The key to this kit is the high sensitivity of detection of Fc-tagged Spike protein by HRP-labeled Anti-Mouse-Fc.

Briefly, ACE2 protein is thawed, diluted 1 μg/mL in PBS, and attached to a nickel-coated 96-well plate at room temperature for 1 h with slow shaking. After the coating, the plate is washed and incubated with a Blocking Buffer for 10 min. Next, 10 μL of inhibitor solution containing the compounds to be tested is added to each well designated Test Inhibitor and incubated at room temperature for 1 h with slow shaking. For the Positive Control and Blank, add 10 μL of inhibitor buffer (5% DMSO solution). Next, SARS-CoV-2 Spike (RBD)-Fc is thawed, diluted to 0.25 ng/μL (approximately 5 nM) in Assay Buffer 1 and added to all wells labeled Positive Control and Test Inhibitor. The reaction is incubated at room temperature for 1 h with slow shaking. After three washes and incubation with a Blocking Buffer (10 min), the plate is treated with an Anti-Mouse-Fc-HRP and incubated for 1 h at room temperature with slow shaking. Finally, an HRP substrate is added to the plate to produce chemiluminescence, which then can be measured using the FluoStar Omega microplate reader.

## Results

### Virtual Screening

In the light of the search for novel and druggable ACE2 activators, we pursued a VS campaign of an in-house library of 67 natural compounds and semisynthetic bile acid derivatives. In particular, we performed molecular docking, which is a method of choice to study ligand/protein binding interaction,^49−52^ on the ligands in the X-ray structure of ACE2 in the open conformation (PDB ID 1R42)^31^ retrieved from the Protein Data Bank. Docking calculations were carried out in the hinge-bending region, previously proposed as ACE2 activator binding site.^21,22^ Accordingly, we included some of the previously identified ACE2 activators, hydroxyzine, minithixen, xanthenone, and DIZE in the VS library.

VS results show ADscores (Table 1) for several BA derivatives comparable, if not better, to those of previously identified ACE2 activators (Supporting Information Table S1).

**Table 1:**
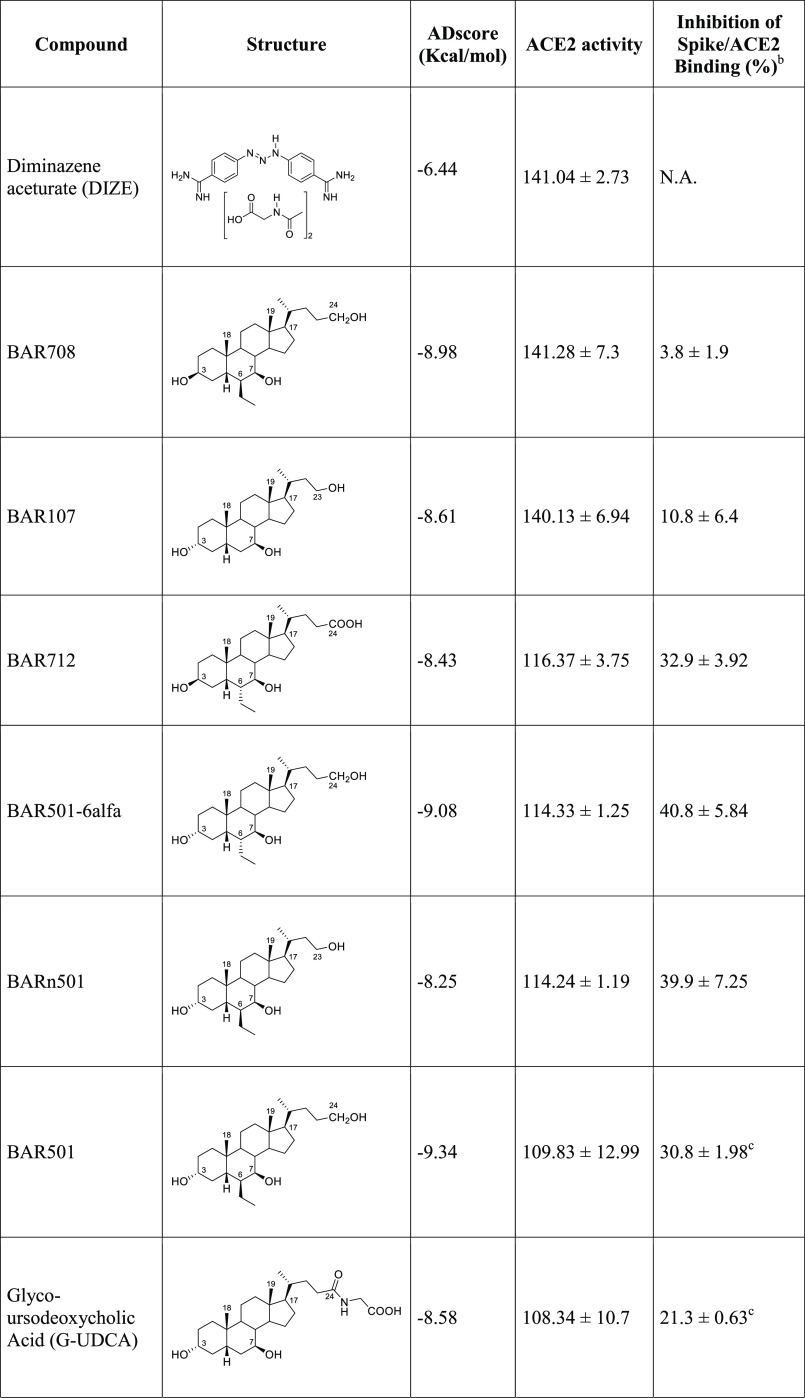

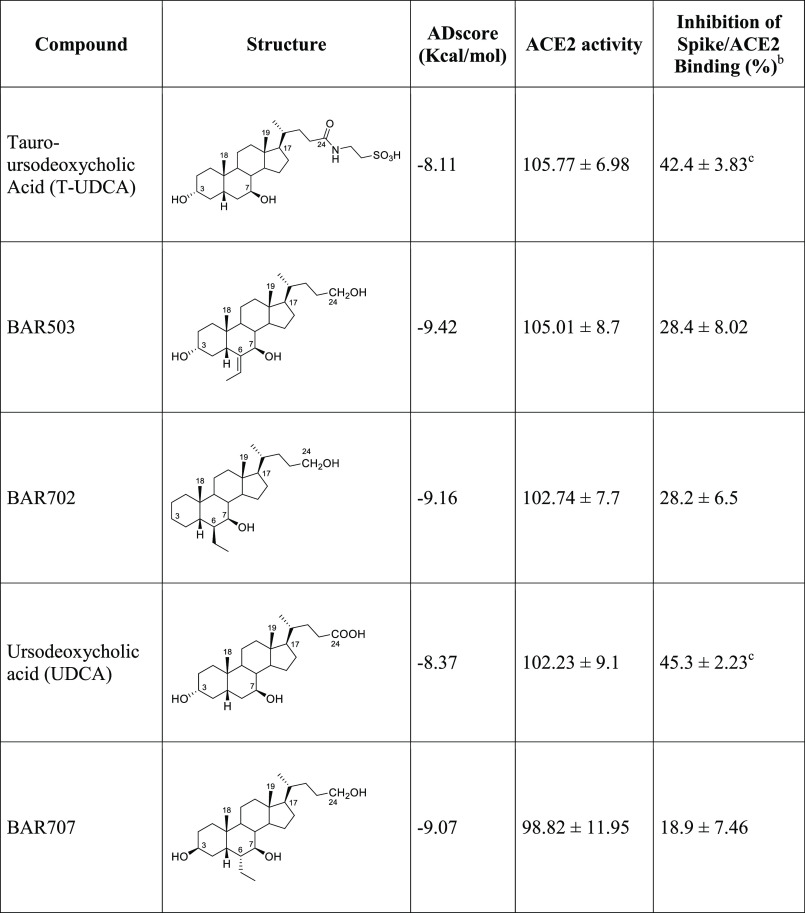
AutoDock4 Docking Scores (ADscore), Enzymatic Activity, and Inhibition of ACE2/Spike Interaction of the Tested Bile Acid Derivatives^a^^,^^b^^,^^c^

a Effect on ACE2 activity of compounds tested at 10 μM, referred to the activity in the absence of any compound (100). Results are expressed as mean ± standard error. *p < 0.05 vs Data are mean ± SE, *n* = 3.

b Inhibition of Spike-RBD/ACE2 Binding for each compound tested at 10 μM, expressed as % ± SE.

c Data taken from ref (35).

Docking results show, for all of the compounds, similar binding modes within the ACE2 hinge-bending region, contacting both residues from Sub I (Lys94, Leu95, Glu98, and Glu102–Helix α3) and Sub II (Tyr202, Asp206 from Helix α7, Val209, Asn210 from Helix 3_10_ H3, Pro565 and Trp566 from Helix α19) (Supporting Information Figure S3; for secondary structures numbering, see Supporting Information Figure S4).

### In Vitro Activity Assays

Prompted by the promising docking results, we investigated experimentally the activity toward ACE2 of the top score compounds (see Table 1, Figure S5). ACE2 activity was assessed using the ACE2 Inhibitor Screening Assay Kit and using DIZE as an ACE2 reference activator. As reported in Table 1, while none of the tested compounds inhibited ACE2 activity, BAR708, BAR712, BARn501, BAR501–6α, and BAR107 significantly increased ACE2 activity (Table 1, *p < 0.05 *n* = 3 replicates), with BAR708 and BAR107 found as effective as DIZE in activating ACE2, thus confirming docking’s prediction about the possibility of UDCA derivatives being able to bind and activate ACE2.

Finally, considering that we have recently discovered the potentiality of BA derivatives to interfere with the ACE2/SARS-CoV-2 RBD region binding process,^35^ we have further investigated whether the compounds mentioned in Table 1 impacted on the binding of Spike-RBD to the ACE2 through a Spike/ACE2 Inhibitor Screening Assay Kit.^35^ Incubating the Spike-RBD with UDCA, T-UDCA, and several UDCA derivatives, the binding of Spike-RBD to ACE2 was effectively reduced in a concentration-dependent manner (Table 1, *n* = 3 replicates). In particular, BARn501 and BAR501–6α were able to inhibit in vitro RBD/ACE2 interaction of ∼40%, while many other compounds of the series inhibited the interaction of ∼30%, thus confirming the previous finding of the suitability of the BA scaffold to inhibit in vitro the RBD/ACE2 interaction.

### ACE2 Structure and Dynamics

With the aim to disclose the mechanism of action of ACE2 activators, we first investigated the structure and the dynamic behavior of the ACE2 metalloproteinase domain (PD domain). We used two X-ray 3D structures of human ACE2 deposited in the Protein Data Bank corresponding to the native (apo) and inhibitor-bound forms of the ACE2 PD (PDB ID 1R42 and 1R4L, respectively).^31^ The two structures represent two different conformational states, an open (native) and a fully closed (inhibitor-bound) state, suggesting a possible equilibrium between the two states, which differ for the relative position of two noncontinuous subdomains, the N-terminal subdomain I (Sub I; residues 19–102, 290–397, and 417–430) and the C-terminal subdomain II (Sub II; residues 103–289, 398–416, and 431–615) (Figure 1A). To establish if ACE2 spontaneously undergoes a conformational equilibrium between these states, three independent molecular dynamics (MD) simulations of 500 ns each of ACE2 apo (PDB ID 1R42) for a total simulation time of 1.5 μs have been performed. All of the conformations visited by ACE2 during the simulations were collected and analyzed through hierarchical clusterization. The cluster analysis showed that the most populated cluster family, accounting for 1/3 of the total population (32%), corresponds to the closed form of the enzyme observed in the crystal structure of the ACE2/inhibitor complex (PDB ID 1R4L). The second (20%) and third (18%) most populated clusters represented a fully open conformation comparable to the crystal structure of the native ACE2 and an intermediate conformation (Figure 1B, Supporting Information Figure S6), respectively.

Successively, essential motions emerging from the three MD simulations were analyzed through principal component analysis (PCA), which allows identifying the largest conformational changes of the enzyme associated with the longest timescale. The PCA analysis computed on the merged trajectory showed the first two main components consisting of movements of Sub I toward Sub II. In the first component (PC1; Figure 1C), the movement of helix α4 and β1−β2 sheets of Sub II toward helix α2 and the loop between α10 and β4 was observed (Supporting Information Video S1), while the second component (PC2) described the approach of helixes α1 and α2 toward the helix 3_10_ H2 (PC2; Figure 1D; Supporting Information Video S2). The PC3 component described a slide motion of Sub II relative to Sub I (PC3; Figure 1E; Supporting Information Video S3). Taken together, these movements describe the conformational equilibrium that should be altered to favor the open ACE2 conformation, enhancing the enzymatic activity. According to the PC1 and PC2, the critical region enabling structural flexibility of ACE2 was delimited by α4, the C-terminal side of α6, and the helix 3_10_ H3. Interestingly, this region is within the hinge-bending region and corresponds to the BAs binding site identified through the docking calculations.

### Mechanism of ACE2 Activation

Prompted by the experimental data obtained for the ligands coming from the VS campaign, we performed an MD simulation of ACE2 in complex with the most active compounds BAR107 and BAR708 using for each system the best ligand pose resulting from the docking calculations as starting conformation (Supporting Information Figure S3B,C, respectively). Three independent MD runs of 500 ns each were carried out for a total simulation time of 1.5 μs. Simulations of each system were merged to analyze the dynamic behavior of the enzyme. The analysis of the merged MD simulations showed a stable and convergent binding mode for BAR107 and BAR708, as shown by the RMSD and SASA evolution over time (Supporting Information Figure S7). Similarly, hierarchical cluster analysis yielded a prevalent binding mode (85 and 76% of the population for BAR708 and BAR107, respectively) (Supporting Information Figure S7E,F). One might note a change in the binding mode of BAR107 in the last 50 ns of the second MD run, in which an inversion of the binding orientation of the ligand at the binding site is observed. However, such conformational change is not statistically relevant—belonging to a less populated cluster (Supporting Information Figure S7D,F)—and it is likely due to the plasticity and solvent-exposed nature of the binding site at ACE2 hinge-bending region. To better investigate changes in the position and conformation of the ligands, their solvent-accessible surface area (SASA) was further calculated. Specifically, the SASA of BAR708 (Supporting Information Figure S7I) has minor fluctuations with respect to the SASA of BAR107 (Supporting Information Figure S7J) in all of the three MD runs, thus highlighting a less tendency to move toward a more solvent-exposed environment.

**Figure 2:**
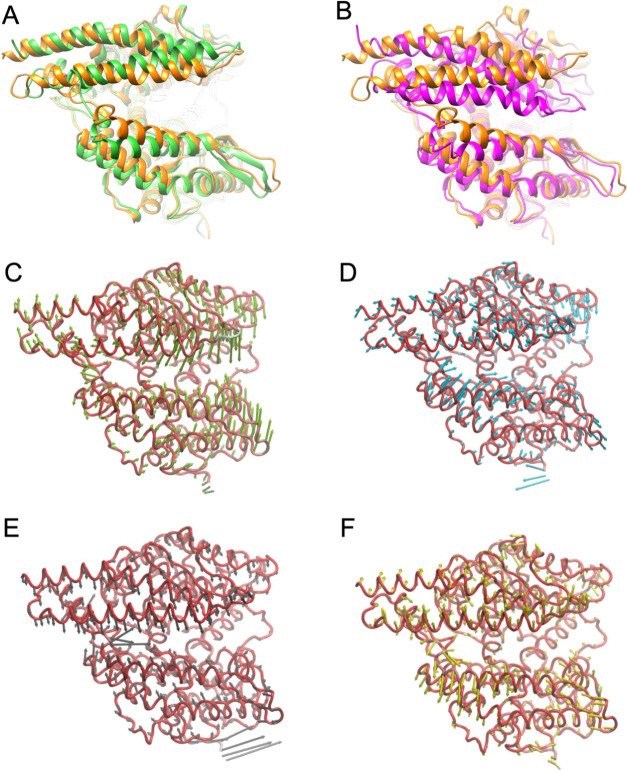
Dynamic states of ACE2 complexed with the activator BAR708. (A, B) Superimposition on the Sub II protein backbone between (A) the most populated cluster over 1.5 μs MD of the BAR708/ACE2 complex (orange cartoon) and the X-ray structure of the open apo ACE2 (PDB ID 1R42; green cartoon) and (B) the most populated cluster over 1.5 μs MD of the BAR708/ACE2 complex (orange cartoon) and the closed ACE2 complexed with the potent inhibitor MLN-4760 (PDB ID 1R4L; magenta cartoon). (C–F) Correlated motions from the PCA analysis during 1.5 μs MD simulations of the BAR708/ACE2 complex, represented by porcupine plots of the first four vectors (PC1–4). Protein backbones are represented as ribbons, the arrows indicate the direction of the motion, and the length represented the magnitude of the corresponding eigenvalue.

Successively, we investigated if the binding of BAR708 and BAR107 influenced the conformational behavior of the apo ACE2 receptor observed in MD simulations. The analysis of the trajectories showed that the binding of BAR708 strongly affects the enzyme dynamics impeding a full closure of Sub I to Sub II. Indeed, the main cluster (85% of the population) of the BAR708/ACE2 complex simulations shows an open structure, very similar to the X-ray open structure of apo ACE2, as shown by the superimposition with the X-ray open and closed forms (Figure 2A,B, respectively). The analysis of the correlated motions of the protein, represented by the top four principal components (PC1–4) of BAR708/ACE2 MD simulations (Supporting Information Videos S5–S8), reveals the same components found in apo ACE2, with a marked reduction of the length of the vector and in a different order. In particular, PC2 represents a slide motion between Sub I and Sub II (Figure 2D; PC3 in apo ACE2 PCA; Supporting Information Video S6), while the PC3 component describes the approach of helixes α1 and α2 toward the helix 3_10_ H2 (Figure 2E; PC2 in apo ACE2 PCA; Supporting Information Video S7). These results highlight that the binding of BAR708 in the hinge-bending region alters ACE2 dynamics, preventing the spontaneous closure of Sub I on Sub II, thus providing evidence of a molecular mechanism of ACE2 activation.

The analysis of the MD simulations of BAR107 further confirms our findings. In particular, the protein clusterization of the BAR107/ACE2 complex yielded the main cluster (76% of the population; Supporting Information Figure S7F), corresponding to an open structure (Supporting Information Figure S8A,B). The analysis of the top four principal components (PC1–4) of BAR107/ACE2 MD simulations (Supporting Information Figure S8C–F, Videos S9–S12) shows the same behavior observed in BAR708/ACE2, with a marked reduction of the closure of Sub I on Sub II.

**Figure 3:**
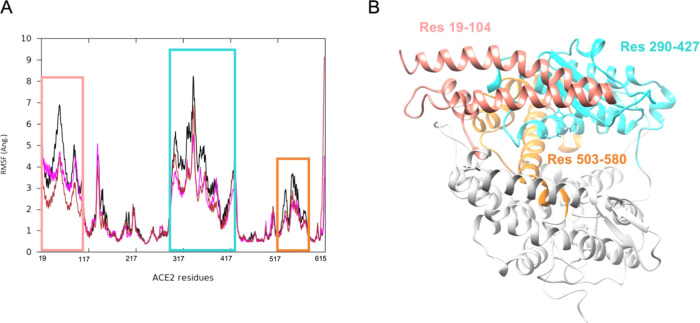
(A) RMSF plot of the ACE2 residues in different systems: ACE2 apo form (black line), ACE2 complexed with BAR708 (brown line) and BAR107 (magenta line). (B) Open native state of ACE2 highlighting the most fluctuating residues from RMSF analysis. RMSF was calculated on Cα atoms aligning on the α-helices of Sub II.

To describe the overall dynamics of the proteins, we also calculated the fluctuation of residues in different systems (RMSF). The results (Figure 3) highlight that binding of the ACE2 activators affected the protein motion in three regions, significantly reducing the amplitude of fluctuation of residues in all of the three regions, compared to the apo ACE2 MD. As expected, apo ACE2 RMSF (black line) shows higher fluctuations compared to the activator-bound complexes (brown and magenta lines).

### Binding Mode of BA Derivatives on ACE2

**Figure 4:**
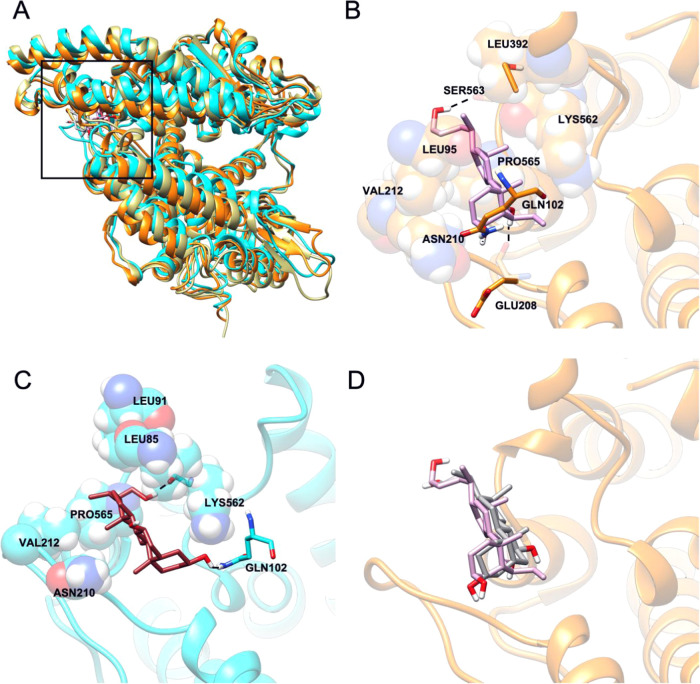
(A) Overall representation of the most populated MD-derived clusters of BAR708/ACE2 complex (orange cartoon) and BAR107/ACE2 complex (cyan cartoon). The black square indicates the hinge-bending region targeted as the agonist binding site. (B) Cluster0 (85%) binding mode of BAR708/ACE2 complex (protein is represented in the orange cartoon, while ligand in the light-violet stick). (C) Cluster0 (76%) binding mode of BAR107/ACE2 complex (protein is represented in the cyan cartoon, ligand in the brown stick). (D) Superimposition between the cluster1 of BAR107/ACE2 complex and the cluster0 BAR708/ACE2 complex (protein represented in the orange cartoon, BAR708 in the light-violet stick, and BAR107 in the dark-gray stick).

The analysis of MD simulations showed a very similar behavior induced by the binding of ACE2 activators, BAR708 and BAR107, to the ACE2 receptor. Both BAR107 and BAR708 interact in a specific binding pocket stacked between the helix α3, the loop between α7 and α8, and the loop connecting α18 and α19, albeit some differences in the pattern of interactions, related to the chemical properties and the position of the substituents on the steroidal scaffold, have been observed (Figure 4).

In particular, the binding mode of BAR708 in ACE2 is rather stable along the three MD simulations carried out for a total of 1.5 μs (Supporting Information Figure S7). The centroid of the most populated cluster (85% of the population) shows the steroidal scaffold located in an amphipathic pocket, allowing contacts with the hydrophobic side chains of Val212 and Leu392 and with the methylene chain of Lys562, while the 3β-OH H-bonds with the side chain of Gln102, whereas the 7β-OH H-bonds with the backbone of Glu208. Also, the ligand side chain at C17 contributes to the binding through hydrophobic contacts with the side chains of Leu395 and Pro565. Finally, the hydroxyl group at the C24 side chain makes an additional H-bond with the side chain of Ser253 (Figure 4B).

On the other hand, the clusterization of the conformations of BAR107/ACE2 complex explored during the MD simulations based on ligand RMSD yielded the main cluster accounting for 75% of the population, and a second populated cluster including 19% of the population (Figure S7F). In the binding mode of the main cluster, BAR107 is placed in the same amphipathic pocket of BAR708, between the helix α3 and the loop between α7 and α8 (Figure 4C). However, at this pose, the ligand is rotated along the axis of the steroidal scaffold compared to BAR708, maintaining the H-bonds between 3α-OH and the side-chain carbonyl of Gln102, and the hydrophobic interaction of the C24 chain with Pro565. Furthermore, the steroidal scaffold makes hydrophobic contacts with Leu95 and the methylene chain of Lys562, while the C21 methyl group interacts with Val212, and, finally, the ligand alkyl chain at C17 engages contacts with Leu91. Moreover, the 3α-OH H-bonds the side chain of Gln102, whereas the C19 methyl group points toward the methylenes of Asn210 and Glu208 side chains. Finally, the hydroxyl group at C24 on the side chain made an H-bond with the backbone carbonyl of Lys562. Notably, the second cluster presents a binding mode very similar to that of BAR708 (Figure 4D) and does not show appreciable differences in protein conformation, as shown by the very low RMSD value computed for the protein backbone (1.8 Å).

## Discussion and Conclusions

**Figure 5:**
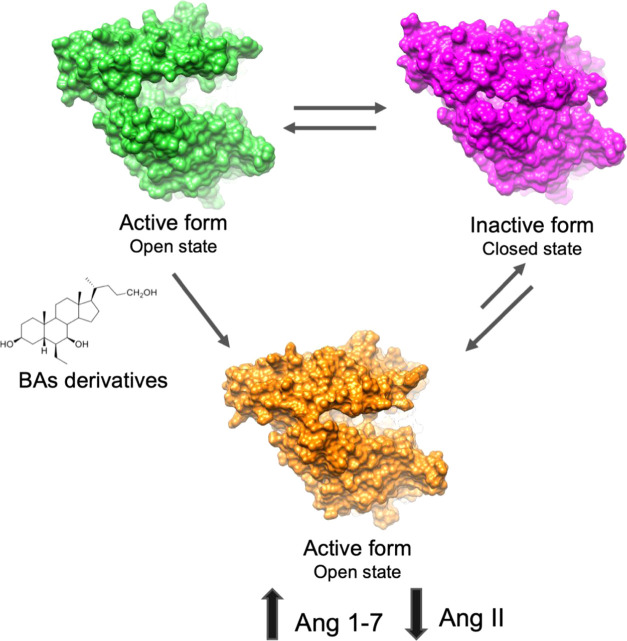
Schematic representation of the proposed mechanism of action.

In the present study, we report the discovery of UDCA derivatives as potent ACE2 activators together with a structural insight into their mechanism of action by means of atomistic MD simulations and investigation of the slowest degrees of freedom of ligand/ACE2 systems (*i.e.*, PCA) (Figure 5).

The interest in ACE2 activation arises from the protective role exerted by this enzyme in different disorders including hypertension and inflammation. The role of ACE2 is twofold: first, it hydrolyzes Ang II, contributing to reduce the activation of RAS; second, the product of the hydrolysis, Ang (1–7), activates the GPCR Mas receptor. MasR activation leads to several biological effects including vasodilation, release of nitric oxide, prostaglandin E2, and bradykinin, with consequent reduction of oxidative stress and inflammation.^11−13^ Moreover, Ang (1–7) counteracts leukocyte migration, cytokine expression and release, and fibrogenic pathways;^11^ decreases the expression of inflammatory mediators including TNFα, IL-1β, IL-6, and MCP-1; and increases the expression of the anti-inflammatory cytokine IL-10.^14−16^ Therefore, ACE2 could be considered as an attractive pharmaceutical target for novel therapeutic approaches to treat hypertension,^24^ myocardial infarction,^25^ liver injury,^26^ kidney disease,^27^ and inflammation.^28^ The interest in the pharmacological activation of ACE2 has been recently also related to the pathological conditions characterizing severe symptoms in COVID-19 patients, due to the anti-inflammatory effects of ACE2 activation. In this scenario, we performed a virtual screening campaign to identify novel bile acid derivatives able to activate ACE2. We screened a database of natural compounds and semisynthetic derivatives targeting the hinge-bending region, followed by *in vitro* enzymatic assays. Our approach led us to identify a series of UDCA derivatives as promising ACE2 activators. In particular, two compounds of the series, BAR107 and BAR708, showed ACE2 activation comparable to that of DIZE, the most investigated ACE2 activator,^23−28^ typically used as a reference compound in enzymatic assays. To disclose the mechanism of activation of ACE2 by BAs derivatives, we first analyzed the conformational dynamics of the apo ACE2 PD domain, through the analysis of 1.5 μs of MD simulations, starting from the crystal structure of native ACE2 (PDB ID 1R42). MD simulations revealed that the open apo state of ACE2 undergoes conformational changes involving the motion of one region (Subdomain I) toward another (Subdomain II), leading to a fully closed structure resembling the conformation of ACE2 in complex with the potent inhibitor MLN-4760 (PDB ID 1R4L) (Figure 5). The principal component analysis (PCA) of ACE2 MD trajectories suggests that a key role in the open/closure movement is played by the segment of the hinge-bending region including helix α3, the loop between α7 and α8, and the loop connecting α18 and α19. Successively, the conformational changes induced by the binding of BAR107 and BAR708 to ACE2 were studied through three MD runs for a total simulation time of 1.5 μs. The analysis of MD simulations of BAR107 and BAR708 complexes through PCA, RMSF, and conformational clusterization evidences that these compounds effectively affect ACE2 dynamics reducing, if not preventing, the full closure of Subdomain I on Subdomain II. In particular, BAR708 and BAR107 reduce the amplitude of the motions observed in the apo ACE2 MD, as shown by both RMSF and porcupine plots resulting from the PCA (Figure 1). On the other hand, the binding mode of the two compounds shows different ligand orientations within the same binding pocket, albeit two common interactions, between the 3α/β-OH and the side chain of Gln102 (bearing to Sub I) and between the side chain of the tetracyclic core of the ligand and Pro565 (Sub II), have been observed. Furthermore, key hydrophobic interactions are engaged with the hydrophobic residues on helix α3 (Leu91 and Leu95; Sub I), and the loop residues between helices α7 and α8 (Sub II), despite involving different faces of the tetracyclic core structure. Therefore, different chemical substituents on the steroidal scaffold (e.g., different stereochemistry of 3-OH or the lack of the 6α-ethyl group in BAR107 with respect to BAR708) might lead to different binding modes that could, however, share common features with similar effects on the ACE2 conformational freedom and eventually leading to comparable activities. We conclude that the intrinsic dynamics of the ACE2 PD domain determine the flexibility of the activators’ binding site that allows the adaptation of BA derivatives endowed with different chemical modifications on the steroidal scaffold. For this reason, defining structure–activity relationships might be very challenging. The series of BA derivatives analyzed in this work differ for: (i) the stereochemistry of the hydroxy group at position 3; (ii) the presence and the stereochemistry of an ethyl group at position 6; and (iii) for the length of the side chain at the C-17 of the tetracyclic core, and finally for the functional end group on the side chain. The analysis of the binding modes of these compounds highlights that the specific stereochemistry of single substituents does not play a key role, and therefore a deeper investigation of the effects of chemical modifications to the steroidal scaffolds on the enzymatic activity is required.

Finally, considering our recent findings on the ability of UDCA and other BA derivatives to affect the interactions between the SARS-CoV-2 spike protein RBD and ACE2 *in vitro*, we have challenged this series of UDCA derivatives in SARS-CoV-2/ACE2 interaction assay and found that two novel BA derivatives, BARn501 and BAR501–6α, inhibit the RBD/ACE2 interaction by ∼40%, while they are mild ACE2 activators. On the other hand, the best ACE2 activators in this series show a very *weak in vitro* inhibition of RBD interaction with ACE2. Interestingly, the reference compound DIZE exerted no effect on the RBD/ACE2 interaction. Our data suggest that UDCA derivatives can bind both ACE2 and the SARS-CoV-2 Spike-RBD. ACE2 activation was not related to the inhibitory capacity of BA derivatives (Table 1), thus suggesting that there is no relationship between ACE2 activation and inhibition of RBD interaction, which rules out the possibility that ACE2 binding and activation interfere with the spike binding site. Very recently, microsecond-scale MD simulations of the full-length ACE2 dimer interacting with glycosylated RBD, in a cellular membrane context, have been reported.^53^ These data demonstrated the high flexibility of the ACE2 homodimer and the high stability of the ACE2/RBD interface. Albeit the authors did not analyze the intrinsic flexibility of the PD domain, their data point out the stability of RBD/ACE2 interaction in a context of high flexibility. This is also in agreement with previous studies on the interaction of SARS-CoV-2 spike protein interaction with ACE2, reporting that ACE2 PD flexibility does not affect the SARS-CoV-2 spike affinity.^54^ Finally, it is noteworthy that none of the UDCA derivatives tested to act as ACE2 inhibitors. Considering the protective effect elicited by ACE2, the inhibition of this enzyme would lead to an increase of the Ang II proinflammatory effect, and therefore would be inappropriate in the design of new COVID-19 therapeutics.

Altogether, this information supports our hypothesis that UDCA derivatives exert their effects binding both the Spike-RBD and ACE2, thus confirming the potentiality of UDCA as a lead scaffold for the development of novel dual-activity antiviral compounds able to reduce the spike/ACE2 interaction and to activate the protective effects elicited by the activation of the Mas axis.

## Supporting Information Available

AD4 scores (kcal/mol) of the known ACE2 activators (Table S1); chemical/physical properties distribution of the 67 compounds belonging to the in-house library (Figure S1); re-docking procedure of AutoDock4 (Figure S2); superimposition of best docking poses of all of the compounds reported in Table 1 (Figure S3 A); best docking pose of BAR708 and BAR107 (Figure S3); sequence and the secondary structure labeling of ACE2 (Figure S4); ACE2:SARS-CoV-2 Spike Inhibitor Screening assay (Figure S5 A) and ACE2 activity assay (Figure S5 B) results; cluster analysis and dynamic states of apo ACE2 (Figure S6); time evolution (μs) of BAR708 and BAR107 RMSD (Figure S7); cluster population of BAR708 and BAR107 after 1.5 μs of MD (Figure S7); time evolution (μs) of the protein RMSD complexed with BAR708 and BAR107 (Figure S7); time evolution (μs) of the solvent-accessible surface area (SASA) of BAR708 and BAR107 (Figure S7); and dynamic states and porcupine plots of the PC1–4 vectors of ACE2 in complex with the activator BAR107 (Figure S8) (PDF)
Supporting 3D structures of ACE2/BAR107 complexes in pdb format (PDB)
Supporting 3D structures of ACE2/BAR708 complexes in pdb format (PDB)
A csv file providing the SMILES of the compounds presented in the manuscript (XLSX)
Supporting animation regarding the PC1–4 of apo ACE2 (Videos S1–S4); ACE2 complexed with BAR708 (Videos S5–S8); and BAR107 (Videos S9–S12) Video S1 (MP4)
Video S2 (MP4)
Video S3 (MP4)
Video S4 (MP4)
Video S5 (MP4)
Video S6 (MP4)
Video S7 (MP4)
Video S8 (MP4)
Video S9 (MP4)
Video S10 (MP4)
Video S11 (MP4)
Video S12 (MP4)

## Supplementary Material

ci1c01126_si_001.pdf

ci1c01126_si_002.pdb

ci1c01126_si_003.pdb

## Notes

3D structures of the ACE2/BAR107 and ACE2/BAR708 complexes are provided in pdb format as Supporting Information. All other data are available upon request.

## Abbreviations Used

ACE2: angiotensin-converting enzyme type II
ACE: angiotensin-converting enzyme
Ang: angiotensin
AT1R: Ang II type I receptor
BAs: bile acids
DIZE: diminazene aceturate
GAFF: general amber force field
GPCR: G-protein-coupled receptor
G-UDCA: glyco-ursodeoxycholic acid
HRP: horseradish peroxidase
IL-1β: interleukin-1β
IL-6: interleukin-6
IL-10: interleukin-10
LGA: Lamarckian genetic algorithm
MD: molecular dynamics
MasR: Mas G-protein-coupled receptor
MCP-1: Monocyte chemoattractant protein-1
MD: molecular dynamics
MMP: matrix metalloproteinase
PCA: principal component analysis
PCs: principal components
PD: peptidase domain
PME: particle Mesh Ewald
RAS: renin-angiotensin system
RBD: receptor-binding domain
RESP: restrained electrostatic potential
RMSD: root-mean-square deviation
RMSF: root-mean-square fluctuations
SASA: solvent-accessible surface area
UDCA: ursodeoxycholic acid
TMPRSS2: transmembrane serine protease 2
T-UDCA: tauro-ursodeoxycholic acid
UDCA: ursodeoxycholic acid
VCAM: vascular cells adhesion molecules
VS: virtual screening
