# Supplementary material for: Discovery of Bile Acid Derivatives as Potent ACE2 Activators by Virtual Screening and Essential Dynamics
Source: J Chem Inf Model. 2021 Dec 16;62(1):196–209. doi: 10.1021/acs.jcim.1c01126 (PMC8691454; doi:10.1021/acs.jcim.1c01126)
Supplement: Supplementary file 1 — ci1c01126_si_001.pdf [file ci1c01126_si_001.pdf]

Supporting Information

DISCOVERY OF BILE ACIDS DERIVATIVES AS POTENT ACE2 ACTIVATORS BY VIRTUAL SCREENING AND ESSENTIAL DYNAMICS

Bianca Fiorillo,[1] Silvia Marchianò,[2] Federica Moraca,[1,3] Valentina Sepe,[1] Adriana Carino,[2] Pasquale Rapacciuolo,[1] Michele Biagioli,[2] Vittorio Limongelli,[1,4] Angela Zampella[1], Bruno Catalanotti,[1,] and Stefano Fiorucci[2]*

[1]Department of Pharmacy, Università di Napoli "Federico II", Via D. Montesano, 49, I-80131 Napoli, Italy

[2]Department of Medicine and Surgery, Università di Perugia School of Medicine, Piazza L. Severi, 1-06132 Perugia, Italy.

[3]Net4Science S.r.l., University "Magna Græcia" of Catanzaro, Campus Universitario "S. Venuta", I-88100 Catanzaro, Italy.

[4]Università della Svizzera italiana (USI), Faculty of Biomedical Sciences, Euler Institute, via G. Buffi 13, CH-6900 Lugano, Switzerland.

*To whom correspondence should be addressed.

Bruno Catalanotti Tel.: +39 081678551 E-mail: bruno.catalanotti@unina.it

Table S1. Autodock4 docking scores (ADscore) of known ACE2 activators.

**Acronym**	**Name**	**ADscore (Kcal/mol)**
**CTX**	minithixen (NSC169899)	-8.62
**XNT**	Xanthenone	-8.5
**HXZ**	hydroxyzine (NSC 169188)	-7.63
**DIZE**	diminazene (NSC357775)	-6.44

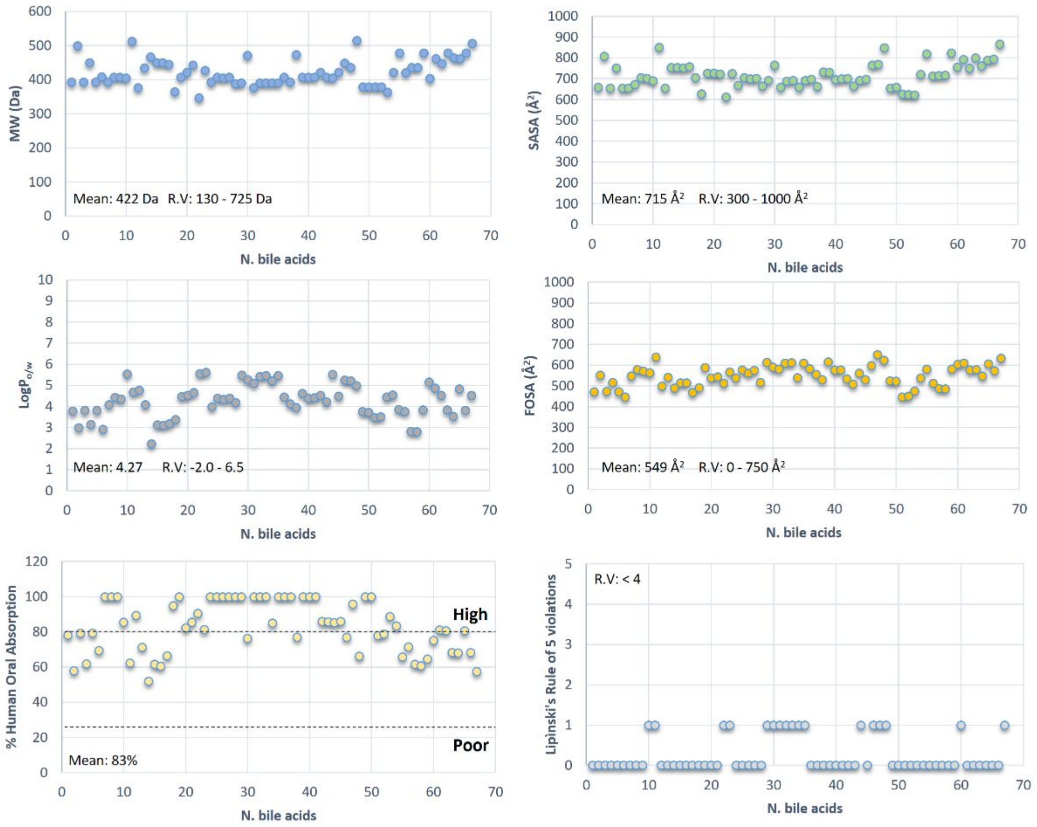

Figure S1: Main chemical/physical properties of the 67 compounds belonging to our in-house library and used as VS database. *R.V is the "recommended value", which is the range belonging to 95% of

known drugs. MW: Molecular Weight; SASA: Solvent-Accessible Surface Area; LogPo/w: predicted octanol/water partition coefficient; FOSA: Hydrophobic component of the SASA.

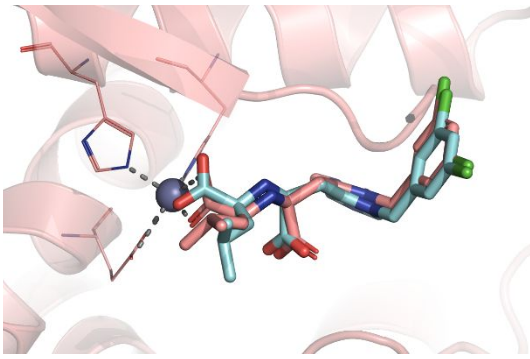

Figure S2: Re-Docking validation procedure performed with the AutoDock4(Zn) force field within the ACE2 antagonist binding site. The AutoDock4(Zn) docking pose of the potent inhibitor MLN-4760 (cyan sticks) is superimposed to the co-crystallographic binding pose (pink sticks). The very low RMSD=1.19Å calculated on the heavy atoms, demonstrates the high performance of this docking algorithm. Hydrogen atoms are omitted for clarity reasons.

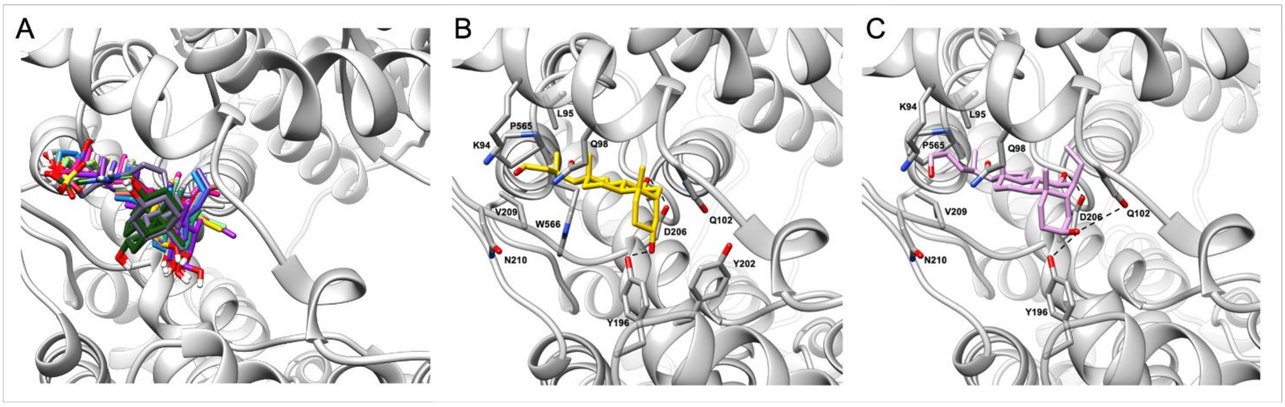

Figure S3. Graphical representation of the binding mode of the best docking poses. (A) Superimposition of the best docking pose for all the compounds in reported Table 1: (B) and (C)

details of the best docking pose of BAR107 (gold sticks) and BAR708 (light-violet sticks), respectively. The interacting residues of the receptor are shown in grey sticks and labelled. Oxygen atoms are depicted in red and nitrogens in blue. Protein receptors are represented as grey cartoon. Hydrogens are omitted for the sake of clarity, while H-bonds are displayed as dashed black lines.

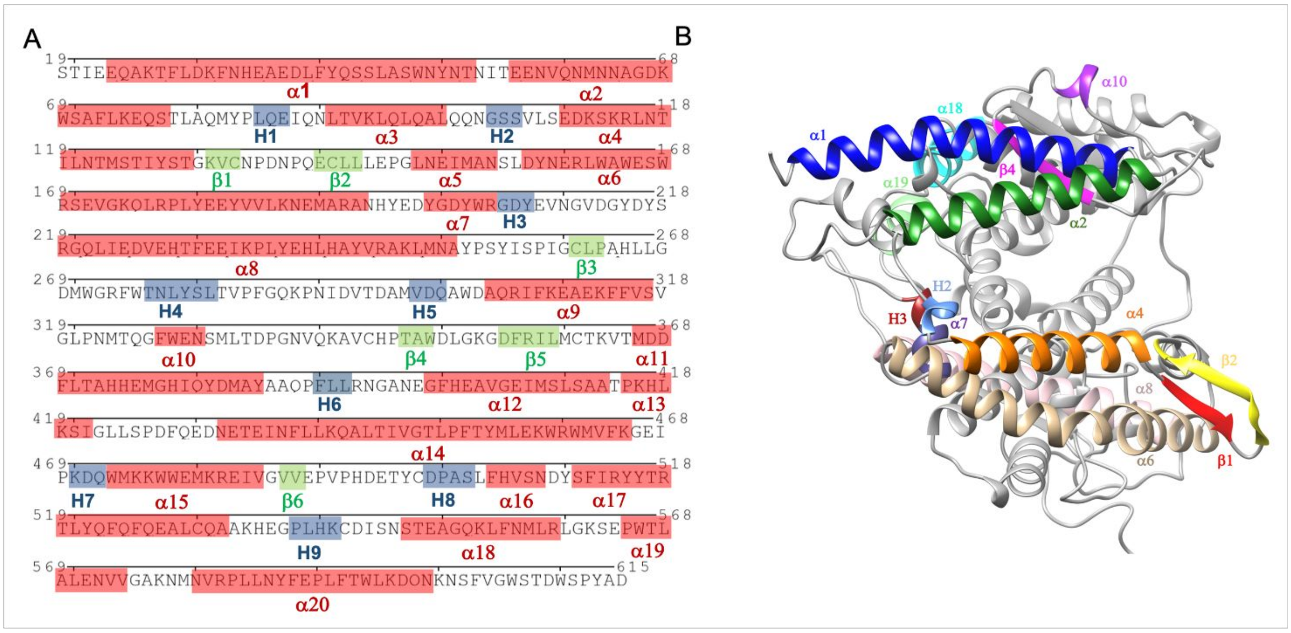

Figure S4. (A) Numbering of the secondary structure of ACE2; (B) Labelling of the more relevant secondary structures in 3D structure 1R42.

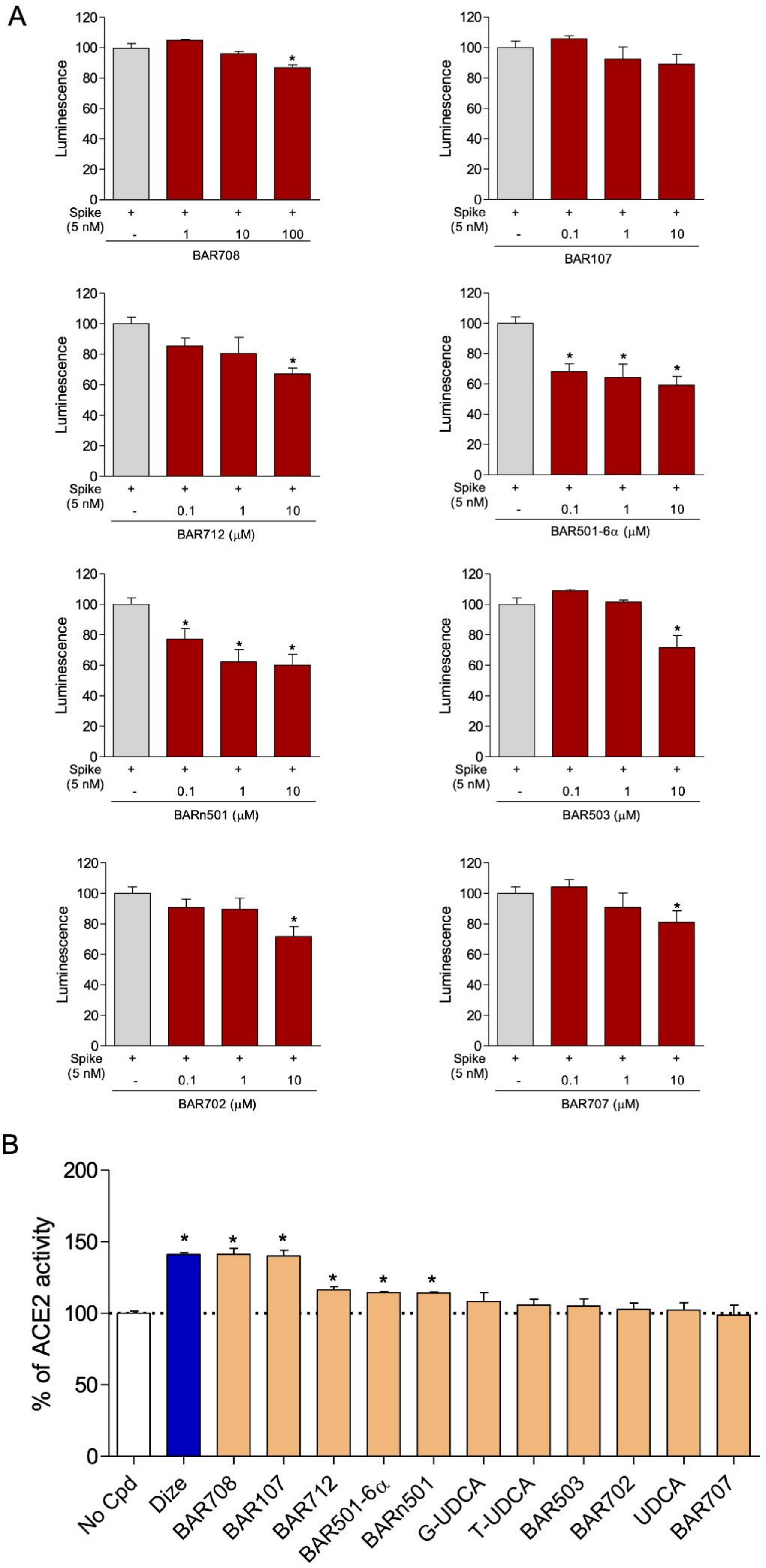
A
Luminescence
Spike
(5 nM)
BAR708
BAR107
BAR712 (μM)
BAR501-6α (μM)
BARn501 (μM)
BAR503 (μM)
BAR702 (μM)
BAR707 (μM)
B
% of ACE2 activity
No Cpd
Dize
BAR708
BAR107
BAR712
BAR501-6α
BARn501
G-UDCA
T-UDCA
BAR503
BAR702
UDCA
BAR707

Figure S5. (A) The ACE2:SARS-CoV-2 Spike Inhibitor Screening assay. BAR708, BAR107, BAR712, BAR501-6α, BARn501, BAR503, BAR702 and BAR707 were tested at different concentration (0.1, 1, and 10μM), to evaluate their ability to inhibit the binding of Spike protein (5 nM) to immobilized ACE2. Luminescence was measured using a Fluo-Star Omega fluorescent microplate reader. Luminescence values of Spike 5 nM were arbitrarily set to 100%. Results are expressed as mean ± standard error. *$p < 0.05$ vs Spike 5 nM. (B) ACE2 activity assay. Compounds were tested on a cell-free enzymatic assay to screen activators of ACE2 activity. Dize was used as positive control. The assay is designed to mesure the exopeptidase activity of ACE2, it utilizes the ability of an active ACE2 to cleave a synthetic fluorogenic substrate to release a free fluorophore. The released fluorophore is quantified using a fluorescence microplate reader. Fluorescence values of activity in absence of any compound were arbitrarily set to 100%. Results are expressed as mean ± standard error. *$p < 0.05$ vs No Cpd.

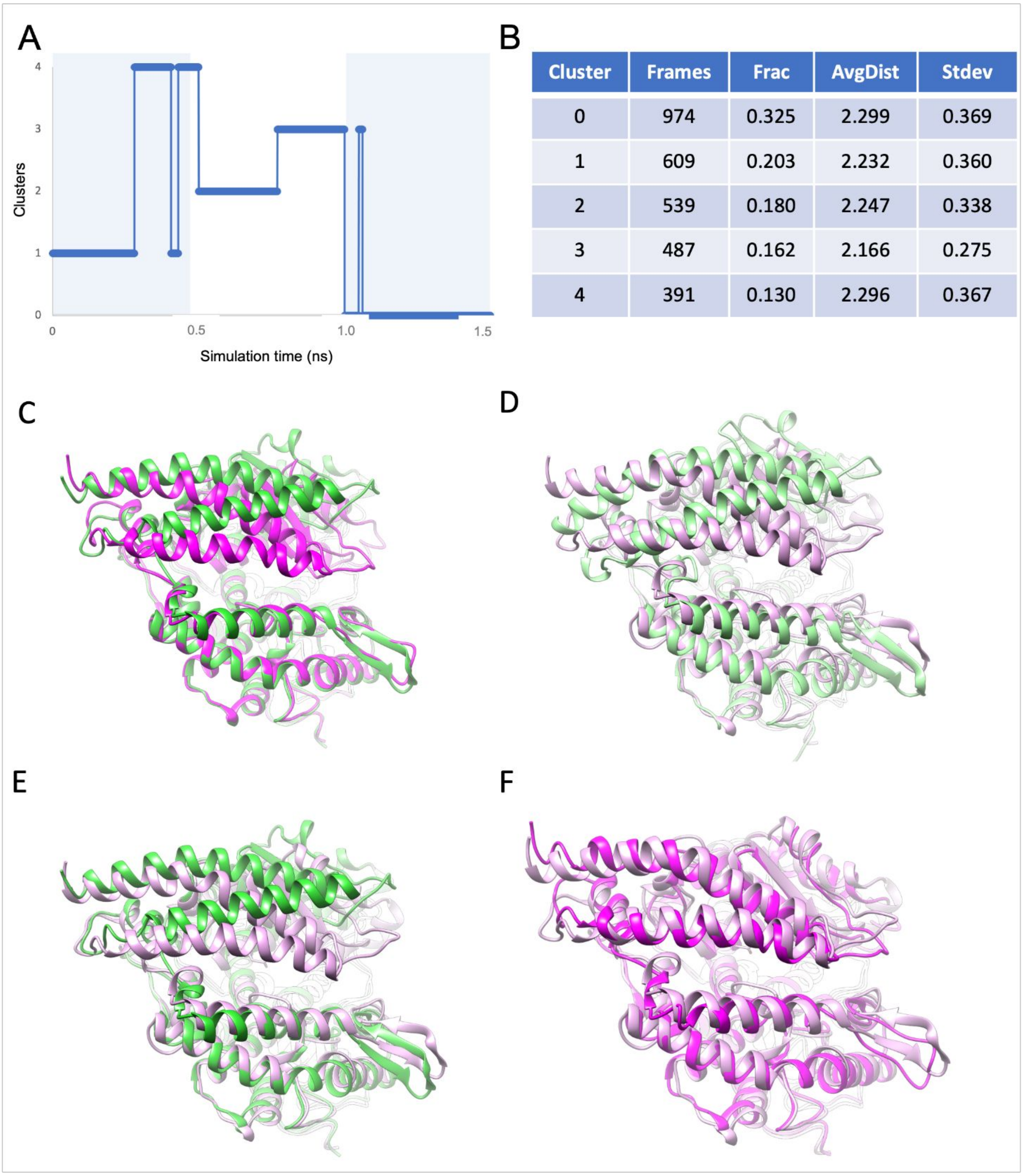

Cluster	Frames	Frac	AvgDist	Stdev
0	974	0.325	2.299	0.369
1	609	0.203	2.232	0.360
2	539	0.180	2.247	0.338
3	487	0.162	2.166	0.275
4	391	0.130	2.296	0.367

Figure S6. Dynamic states of native ACE2. (A) Cluster analysis of the MD simulations of apo ACE2; (B) plot representation of cluster distribution versus MD simulation time of the apo ACE2 receptor. (C-E) Superimposition on the Sub II protein backbone between: (C) the X-ray structures of the open apo ACE2 (PDB ID 1R42) (green cartoon) and the closed state ACE2 in complex with the potent

inhibitor MLN-4760 (PDB ID 1R4L) (magenta cartoon); (D) the cluster1 (light-green cartoon) and the cluster0 (light-violet cartoon) of the apo ACE2 obtained after 1.5 μs of MD; (E) the X-ray structure of the open state apo ACE2 (PDB ID 1R42) (green cartoon) and the cluster0 of the closed form obtained after MD (light-violet cartoon); (F) the X-ray structure of the inhibitor-bound closed form (PDB ID 1R4L), (magenta cartoon), and the cluster0 of the closed obtained after MD (light-violet cartoon).

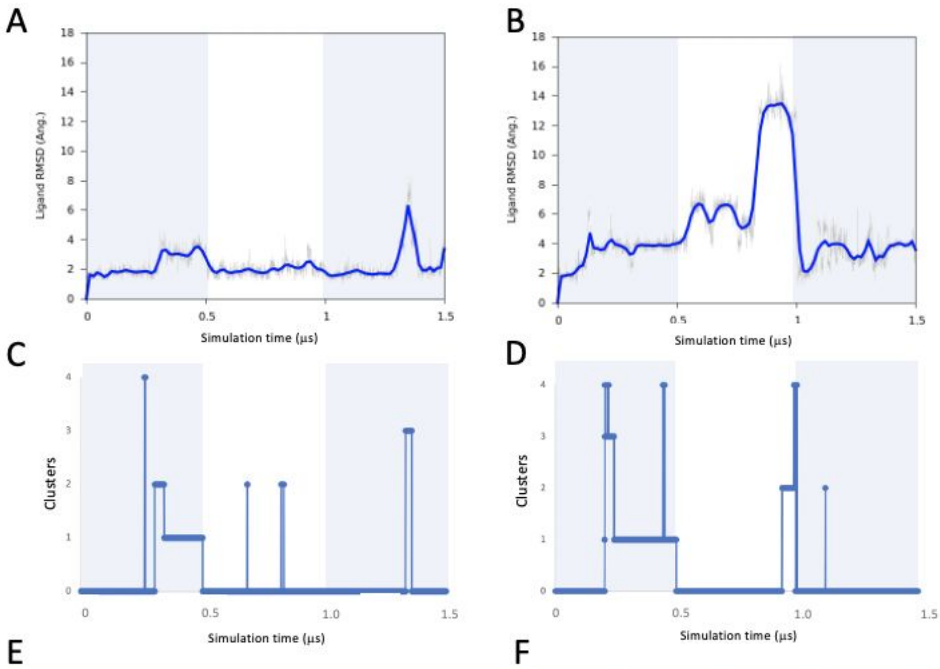

E

Cluster	Frames	Frac	AvgDist	Stdev
0	2542	0.847	1.876	0.357
1	317	0.106	1.757	0.295
2	82	0.027	1.750	0.283
3	51	0.017	1.535	0.231
4	8	0.003	1.329	0.208

F

Cluster	Frames	Frac	AvgDist	Stdev
0	2284	0.761	0.804	0.230
1	505	0.168	0.648	0.234
2	105	0.035	0.570	0.157
3	73	0.024	0.792	0.186
4	33	0.011	0.842	0.216

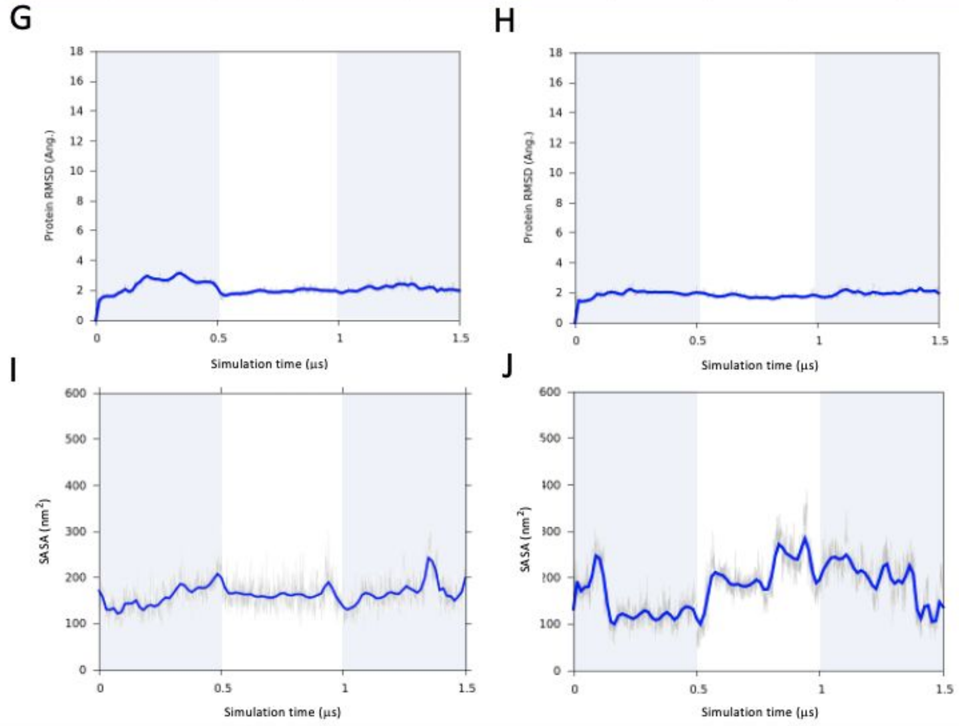

Figure S7. MD evolution time (µs) of the ligand RMSD (Å) of BAR708 (A), BAR107 (B) in ACE2. Plot representation of the cluster population of BAR708 (C) and BAR107 (D) during 1.5 µs of MD simulation. MD evolution time (µs) of the RMSD (Å) of ACE2 complexed with BAR708 (G) and

BAR107 (H). MD evolution time (μs) of the Solvent Accessible Surface Area (SASA) of BAR708 (I) and BAR107 (J) in ACE2 receptor.

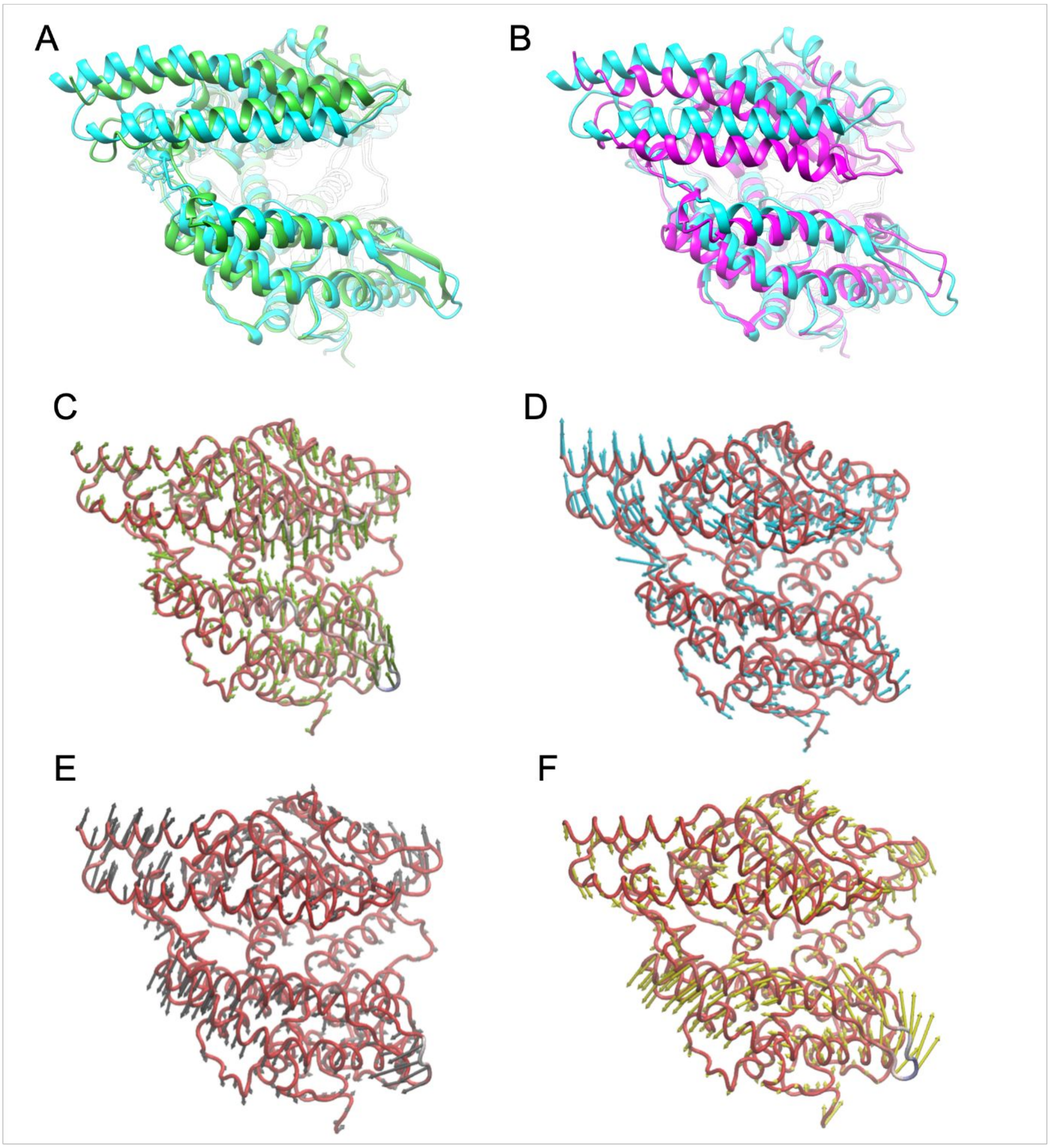

Figure S8. Dynamic states of ACE2 in complex with the activator BAR107: (A) superimposition between the most populated cluster0 (76%) over 1.5 μs MD of the ACE2/BAR107 complex (cyan

cartoon) and the X-ray structure of the open apo form ACE2 (PDB ID 1R42; green cartoon); (B) the most populated cluster0 (76%) over 1.5 μs MD of the ACE2/BAR107 complex (cyan cartoon) on the closed state ACE2 in complex with the potent inhibitor MLN-4760 (PDB ID 1R4L; magenta cartoon); (C-F) Correlated motions obtained from the PCA analysis of 1.5 μs MD simulations of the ACE2/BAR107 complex, represented by porcupine plots of the first 4 vectors. Protein backbones are represented as red ribbons, the arrows indicate the direction of the motion, and the length represented the magnitude of the corresponding eigenvalue.
